# Preimplantation Genetic Diagnosis in Marfan Syndrome

**DOI:** 10.1155/2013/542961

**Published:** 2013-05-27

**Authors:** N. F. Vlahos, O. Triantafyllidou, N. Vitoratos, C. Grigoriadis, G. Creatsas

**Affiliations:** 2nd Department of Obstetrics and Gynecology, “Aretaieion” Hospital, University of Athens, Vas. Sofias 76, 11528 Athens, Greece

## Abstract

Marfan syndrome (MFS) is a systemic hereditable disorder of the connective tissue with mainly cardiovascular manifestations, such as aortic dilatation and dissection. We describe a case of a 32-year-old Caucasian woman, clinically asymptomatic with MFS who presented for genetic consultation to prevent the transmission of disease to her offspring. She underwent controlled ovarian stimulation (COH), in vitro fertilization (IVF) combined with preimplantation genetic diagnosis (PGD), and a singleton pregnancy with positive fetal heart rate was revealed. At 34 weeks' gestation she delivered vaginally a healthy premature male infant weighting 2440 gr. The patient remained asymptomatic during pregnancy, delivery, and 3 months postpartum. It is has to be mentioned that the availability of PGD is essential to prevent the transmission of disease to the next generation.

## 1. Introduction

 Marfan syndrome is an autosomal dominant disorder of the connective tissue with multisystem clinical manifestations. The estimated prevalence of Marfan syndrome is about one case per 5000 individuals, although this figure is probably underestimated due to difficulties in complete recognition of all affected individuals [1]. MFS affects both genders equally without predilection for any particular race or geographical background. The spectrum of defects related to the syndrome is broad, including cardiovascular, ocular, pulmonary, skin, and skeletal systems. The diagnosis is based on the revised 1996 Ghent criteria and relies on physical examination, history, and genetic test [2].

 MFS is caused by a mutation in the fibrillin 1 gene (FBN1) which is localized to the chromosome 15q21 and encodes the extracellular matrix protein fibrillin-1. The FBN1 is a big gene, spanning 235 kb of genomic DNA and composed of 65 exons. Molecular testing for the FBN1 mutation is neither sensitive nor specific for MFS, in part because of size of the gene, the heterogeneity of mutations discovered, the lack of mutational hot spots, and the absence of efficient molecular diagnostic test that offers a highly sensitive mutation detection rate of FBN1. Moreover, while the disorder segregates as a dominant trait in families, about 25% of the cases are sporadic due to de novo mutations and a family history of MFS is not always present as an obvious risk factor [3].

 De Paepe et al. [2] mentioned that eighty percent of patients with MFS have some cardiovascular involvement, ranging from aortic dilatation and dissection (mainly of the ascending part), aortic regurgitation to mitral and tricuspid prolapse with or without regurgitation. Cardiovascular complications are the main cause of morbidity and mortality in patients with Marfan syndrome. Aortic dissection, aortic rupture, and cardiac failure are the main causes of death in these patients especially during their pregnancy [4].

## 2. Case Presentation

 A 32-year-old Caucasian woman requested preconceptional counseling on account of her family history of MFS. Her brother had been diagnosed with the syndrome several years ago and he had recently undergone a successful surgery for aortic aneurysm. He was found positive for a heterozygous nonsense FBN1 mutation 2049C > A (C683X). The patient was clinically asymptomatic and her detailed physical examination by internist, cardiologist, and ophthalmologist did not reveal any signs of the disease. She had genetic consultation and testing and she was also found to be a carrier of the same mutation. Standard tests were carried out (complete blood account, biochemistry) as well as echocardiography and MRI of the heart and large vessels. There were no cardiovascular abnormalities in the patient and the diameter of ascending aorta at the level of Valsalva sinus was 34 mm. She was advised to undergo controlled ovarian stimulation, in vitro fertilization (IVF) combined with preimplantation genetic diagnosis (PGD) to conceive a healthy child. From our perspective we had a comprehensive discussion with the patient regarding the risks of ovarian stimulation as well as the risks of a subsequent pregnancy should it occur. The patient was aware of all the risks associated with the procedure but she was willing to proceed.

 She underwent controlled ovarian stimulation according to a short GnRH antagonist protocol. Eighteen oocytes were retrieved and underwent intracytoplasmic sperm injection (ICSI). Thirteen oocytes were fertilized. Out of those, 9 embryos proceed to the 8-cell stage (day 3 after retrieval) and underwent embryo biopsy. On the 9 blastomeres obtained, genomic DNA was used for PCR amplification of exon 16 in the FBN1 gene, with primers designed to detect the nonsense FBN1 mutation 2049C > A (C683X). The corresponding PCR product was sequenced in both the forward and reverse orientations. Three embryos were identified with the nonsense FBN1 mutation 2049C > A (C683X). Five embryos had inconclusive diagnosis and there was only one apparently healthy embryo. This embryo was transferred at the blastocyst stage (day 5 after retrieval). Two weeks later she had a positive pregnancy test and 4 weeks after the transfer she had a transvaginal sonogram which revealed a singleton pregnancy with positive fetal heart rate. 

 During the course of her pregnancy she had frequent obstetrical visits as well as with her cardiologist. Every trimester she had echocardiographic evaluation of the heart and large vessels which remained unchanged throughout the pregnancy. The aortic diameter did not exceed 27 mm and the sinus of Valsalva 35 mm without any other cardiovascular pathology. She remained hemodynamically stable throughout her pregnancy with blood pressure measurements of 110 to 90 mmHg for the systolic and 65–80 for the diastolic. At 20 weeks of gestation she was diagnosed with decreased cervical length of 18 mm and she underwent a successful cervical cerclage (Shirodkar). At 28 weeks she was diagnosed with gestational diabetes mellitus which was subsequently controlled with SQ-insulin. She had also developed iron-deficiency anemia (hemoglobin: 9.0 g/dL, hematocrit: 28.4%) not responding to oral iron supplementation and she was treated with intravenous iron therapy according to the protocols of our department. At 34 weeks' gestation she was admitted with premature contractions. The cerclage was removed. Epidural anesthesia was administered for pain control. She eventually delivered vaginally a healthy premature male infant weighting 2440 gr. with Apgar scores 7 in 1st minute and 10 in 5th minute. The patient had an uncomplicated postpartum course and she was discharged home on postpartum day 3. The infant was admitted to the NICU for observation and underwent genetic testing for disease. There is no mutation revealed and the infant was discharged after 10 days. The patient remained asymptomatic after delivery and she had another echocardiographic evaluation at 4 weeks and 3 months postpartum with no significant changes.

## 3. Discussion

 Over the course of a normal pregnancy, profound hemodynamic changes such as increase in blood volume, cardiac output, heart rate, left ventricular stroke work, and oxygen consumption occur. In patients with structural abnormalities of the cardiovascular system such as those with MFS these changes may pose significant risk. The most serious complication in those patients is aortic dissection. A thorough clinical assessment is essential in order to estimate the maternal and fetal risk during pregnancy. The primary focus of this assessment should be to evaluate the patient's ability to tolerate the hemodynamic changes described above. The risk factors for pregnancy-associated dilatation or dissection in patients with Marfan syndrome include a large sinus of Valsalva, rapid growth of the sinus of Valsalva during pregnancy, moderate to severe aortic valve or mitral valve regurgitation, and a family history of sudden death or aortic dissection [5]. Katsuragi et al. in their investigation of 28 consecutive Japanese pregnant patients with Marfan syndrome found that a large sinus of Valsalva (>40 mm) at the beginning of the pregnancy posed a significant risk for dilatation or dissection during pregnancy and in the immediate postpartum period [6]. These findings are consistent with previous studies that suggested an expected rate of aortic dissection of 1% and 10% in high-risk patients (aortic root diameter >40 mm, rapid dilatation, or previous dissection of the ascending aorta) but reported a favorable maternal and fetal outcomes during pregnancy when there was minimal aortic enlargement (<40 mm) [7].

 Based on most series, aortic dissection occurred in MFS women in their third decade of life with an average life expectancy of only 32 years; therefore it is advisable to plan a pregnancy at a younger age. Preconceptional counseling should focus on the impact of pregnancy in both the mother and child. Patients should also be informed about the possibility of prenatal diagnosis, using both genetic and fetal echocardiography [5].

Severe expression of the syndrome can occur in an offspring of a mother with a relatively mild symptomatology. Therefore, the significance of PGD for those patients desiring to conceive cannot be overemphasized. The first case of mutation-based prenatal diagnosis for MFS was reported by Rantamaki in 1995 [8] and since then several preimplantation genetic diagnoses have been reported. Loeys et al. [9] completed 15 prenatal and/or preimplantation genetic diagnoses (PGD) in nine families and data from linkage analyses were used in four families. PGD represents an alternative to prenatal diagnosis and allows selection of unaffected in vitro fertilization embryos to establish pregnancies in couples at risk of transmitting a genetic disorder. Despite the significant advantage provided by PGD there are still technical limitations. There are different techniques which are useful for overcoming the problem of insufficient genomic DNA in PGD, reducing the workload of the genetic diagnostic laboratory as well as the average waiting time for patients and increasing the reliability of the diagnosis [10, 11]. Early 3rd trimester fetal echocardiography may assist in the diagnosis of cardiac manifestations of MFS, such as atrioventricular valve regurgitation and dilatation of the aortic root and pulmonary artery. Gestational complications due to the syndrome such as aortic dissection in the mother carry substantial risks to the fetus and should be detected as early as possible. Serial echocardiography evaluations should be performed regularly at the duration of the pregnancy and up to 3 months postpartum. MFS has also been associated with a higher rate (40%) of obstetric complications, such as cervical incompetence, mainly because of premature rupture of membranes, and premature delivery leads to increased infant morbidity and mortality [5].

In patients with MFS who have no cardiovascular involvement and stable aortic diameter (<40 mm), like our patient, vaginal delivery is preferable and cesarean section should be performed only for obstetrical indications. Epidural anes-thesia should be used to minimize pain and stress of labor. 70% of women with MFS present lumbosacral dural ectasia; therefore an anesthesiology consultation is essential prior to delivery. Cesarean section with epidural or general anesthesia is preferred in patients with aortic diameter >4 cm or with progressive dilatation of aorta during pregnancy to minimize the risk of aortic dissection. In cases where the aortic root diameter exceeds 5 cm at the later stages of pregnancy, cesarean section is recommended followed by heart surgery as soon as possible [12].

 Resent advances have led to improved survival and function of patients with MFS. Women with MFS can expect to tolerate pregnancy and delivery well, providing close surveillance by an obstetrician and a cardiologist. The availability of PGD practically eliminated the risk of genetic transmission and has offered to those patients the possibility to procreate and produce healthy children. 
